# Modelling herd immunity requirements in Queensland: impact of vaccination effectiveness, hesitancy and variants of SARS-CoV-2

**DOI:** 10.1098/rsta.2021.0311

**Published:** 2022-10-03

**Authors:** Paula Sanz-Leon, Lachlan H. W. Hamilton, Sebastian J. Raison, Anna J. X. Pan, Nathan J. Stevenson, Robyn M. Stuart, Romesh G. Abeysuriya, Cliff C. Kerr, Stephen B. Lambert, James A. Roberts

**Affiliations:** ^1^ Brain Modelling Group, QIMR Berghofer Medical Research Institute, Brisbane, QLD 4006, Australia; ^2^ Department of Mathematical Sciences, University of Copenhagen, DK-2100 Copenhagen, Denmark; ^3^ Burnet Institute, Melbourne, VIC 3001, Australia; ^4^ Institute for Disease Modeling, Bill and Melinda Gates Foundation, Seattle, WA 98109, USA; ^5^ National Centre for Immunisation Research and Surveillance for Vaccine Preventable Diseases, Westmead, NSW 2145, Australia

**Keywords:** COVID-19, agent-based modelling, Australia, COVID-19 vaccination, Omicron variant, herd immunity threshold

## Abstract

Long-term control of SARS-CoV-2 outbreaks depends on the widespread coverage of effective vaccines. In Australia, two-dose vaccination coverage of above 90% of the adult population was achieved. However, between August 2020 and August 2021, hesitancy fluctuated dramatically. This raised the question of whether settings with low naturally derived immunity, such as Queensland where less than 0.005% of the population is known to have been infected in 2020, could have achieved herd immunity against 2021’s variants of concern. To address this question, we used the agent-based model Covasim. We simulated outbreak scenarios (with the Alpha, Delta and Omicron variants) and assumed ongoing interventions (testing, tracing, isolation and quarantine). We modelled vaccination using two approaches with different levels of realism. Hesitancy was modelled using Australian survey data. We found that with a vaccine effectiveness against infection of 80%, it was possible to control outbreaks of Alpha, but not Delta or Omicron. With 90% effectiveness, Delta outbreaks may have been preventable, but not Omicron outbreaks. We also estimated that a decrease in hesitancy from 20% to 14% reduced the number of infections, hospitalizations and deaths by over 30%. Overall, we demonstrate that while herd immunity may not be attainable, modest reductions in hesitancy and increases in vaccine uptake may greatly improve health outcomes.

This article is part of the theme issue ‘Technical challenges of modelling real-life epidemics and examples of overcoming these’.

## 1. Introduction

Vaccination is the main pathway out of the COVID-19 pandemic towards a setting of controlled endemic disease. Safe and effective vaccines are currently being rolled out worldwide [1,2]. However, delivery rates are highly variable both between and within countries. Australia, like several countries that had success in limiting initial waves of infections in 2020, had low levels of natural immunity from infections up until late 2021. For its initial vaccine roll-out, Australia used two vaccines—BNT162b2 (Pfizer–BioNTech) [3] and ChAdOx1 (Oxford–AstraZeneca) [4]—with mRNA-1273 (Moderna) [5] and NVX-CoV2373 (Novavax) [6] added to the national schedule in the final quarter of 2021.

Furthermore, Australian state and territory governments linked relaxation of public health measures to reaching two-dose vaccination targets of 70%, 80% and 90% of the eligible population. For instance, New South Wales enacted several policy relaxations upon reaching the 70% target, including, but not limited to, reopening hospitality venues, recreation and sporting facilities, increasing the number of people that can attend weddings, funerals and places of worship, and resuming within-state travel; Queensland nominated the date interstate borders would reopen based on an estimate of when the 80% target would be achieved; and Tasmania implemented a risk-based home quarantine system upon achieving the same 80% target, but reopened the borders upon achieving a 90% target for an eligible population that included people aged 12 years and older.

However, important questions remain about the level of protection offered by different levels of vaccination coverage in the face of variants of concern and age-dependent hesitancy among the population.

An important immunity state is that of ‘herd immunity’, that is, resistance to the spread of an infectious disease within a population that is based on pre-existing immunity of a high proportion of individuals. In the absence of widespread natural immunity, achieving such a state relies on widespread vaccine uptake, with the unvaccinated subset of the population being conferred protection by proxy. Ideally, this unvaccinated subset would only consist of members of the population who are unable to receive the vaccine due to medical or health-related reasons. In practice, idealized herd immunity for SARS-CoV-2 is likely to be difficult to reach, as supported by various models [7–19]. Thus, it is important to determine whether there is a ‘practical’ vaccination coverage level at which introduced infections do not lead to outbreaks, or small outbreaks can be effectively suppressed with only mild additional public health interventions such as testing, contact tracing, isolation and quarantine (TTIQ).

One of the key challenges to herd immunity is vaccine hesitancy and resistance [20–23]. For COVID-19, in particular, evidence of the ChAdOx1 (Oxford–AstraZeneca) vaccine causing very rare clotting disorders with low platelets [24] coincided with increased hesitancy and resistance rates in early 2021 compared with late 2020, particularly in people younger than 60 [25,26]. While Australia eventually achieved very high (>90%) two-dose coverage in December 2021, it remains important to understand the extent to which variable rates of hesitancy, both overall and in an age-specific manner, could have left the Queensland population at risk of outbreaks after completing the vaccine roll-out.

The other key challenge to herd immunity is the emergence of new variants of SARS-CoV-2. In Australia, the most prominent two variants in 2021 were the Alpha (B.1.1.7) and Delta (B.1.617.2) variants, with the arrival of Omicron (B.1.1.529) in December 2021. Since the beginning of the pandemic in early 2020, up until November 2021, Queensland had successfully suppressed small incursions of both Alpha and Delta despite the ongoing risk from outside the state. This suppression capability was achieved through a combination of interventions, including testing, tracing, quarantining, mask wearing and short but widespread lockdowns. However, with the relaxation of an elimination policy, these interventions were shown to be insufficient to stop the spread of either Delta (modelling study [27]) or Omicron (empirical data [28,29]). The increased outbreak risk of more infectious variants was clear from earlier modelling studies [30–37] and has been borne out in outbreaks globally, including in Australia [7,10,12,14,37–40]. The Delta variant is characterized by several mutations that result in increased replication, higher viral load and increased transmission [41] relative to the Alpha variant and the ancestral strain. The vaccines available in Australia have different levels of effectiveness depending on the variant. For instance, the Oxford–AstraZeneca and Pfizer–BioNTech vaccines showed only a small decrease in effectiveness in prevention of symptomatic COVID-19 from Alpha to Delta (from 74.5% to 67.0% and from 93.7% to 88%, respectively [41]), but a large drop in effectiveness against Omicron (25–70% at peak across all vaccines [42]). Moreover, vaccine effectiveness wanes over time [43,44]. On the other hand, two doses of either vaccine exhibit high effectiveness (greater than 90% even after several months) against severe or critical illness and death, for both the Alpha and the Delta variants [42,44]. The rise of the Omicron variant and its subvariants, driven largely by immunity escape, illustrates the importance of understanding the effects of varying vaccine effectiveness on population-level dynamics.

In this article, we examine constraints on achieving effective herd immunity in settings with low natural immunity, including the effects of vaccine hesitancy and variants of concern. While our simulations focus on the state of Queensland, Australia, our results are applicable to other settings with similar demographics and low natural immunity.

We recognize that the generation and obsolescence of knowledge on SARS-CoV-2 proceed at a fast pace. Thus, we note that the models used in this work were implemented between June and August 2021, and wherever possible we used input data available in September 2021. The main text and results were written between August and October 2021 and updated in April 2022 to include the Omicron variant.

## 2. Methods

### (a) Model description

We used an agent-based model, Covasim [31,45], calibrated to the setting of Queensland, Australia [33]. To predict the transmission of SARS-CoV-2 between individual people within the partially vaccinated Queensland population, we modelled this population of approximately 5.2 million people with 200 000 agents, representing a population scale of 26 people per agent. The choice of the number of agents reflected a trade-off between maximizing realism (i.e. by minimizing the number of people per agent as much as possible, as suggested by the developers of Covasim) and ensuring that it would be practical to run millions of simulations given our computational resources.

**Figure 1.**
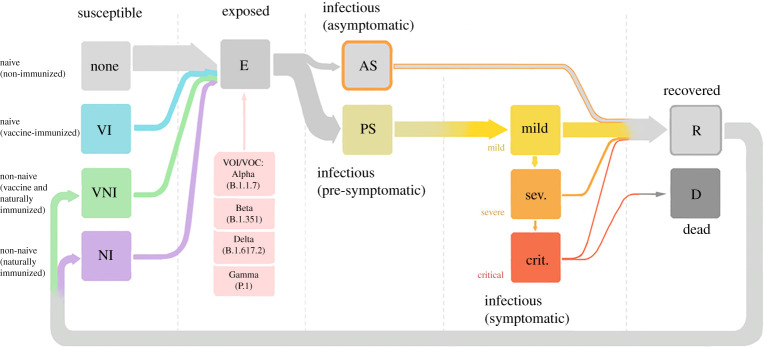
Schematic illustrating the mutually exclusive states agents can take in the model. Arrows indicate direction of disease progression. Pink boxes denote variants of concern to which an agent can be exposed and whose properties (such as transmissibility and severity) are already implemented in Covasim v. 3.0.7 and above [31]. In this work, however, we only study the effects of the Alpha (B.1.1.7) and Delta (B.1.617.2) variants. (Online version in colour.)

In the model, agents transition between states in a probabilistic manner, following the introduction of infections. Agents can be susceptible, exposed, infectious, recovered or dead, with the paths of disease progression or recovery depicted in figure 1. Agents that have been infected can become susceptible and be reinfected (indicated by the path back from recovered to susceptible), though their susceptibility will be lower than a completely naive agent. To reflect the current heterogeneity of the population in terms of susceptibility and immunity, we model susceptible agents as being in one of four different states, depending on whether they have received the vaccine and whether they have been previously infected: naive (never infected) and unvaccinated (grey icon); naive and vaccinated (light blue icon); non-naive and vaccinated (green icon); and non-naive and unvaccinated (violet icon). Agents can be *exposed* to any of the current variants circulating and become *infectious*. From this exposed state, a proportion of agents will be infectious and develop symptoms (symptomatic) of increasing severity (mild, severe and critical), or will not develop symptoms at all (asymptomatic). From any of the symptomatic states, the agents can recover. However, agents that are in a critical symptomatic state, which we take as representative of requiring treatment in an intensive care unit (ICU), can either recover or die. Recovered agents go back to the susceptible state. The probability that a given agent is infected with COVID-19 depends on interactions between the agent and all their contacts across several social networks [33,38,45]. As in the previous work [33], we modelled 14 different social networks including households, schools, workplaces and transport.

### (b) Input data

**Figure 2.**
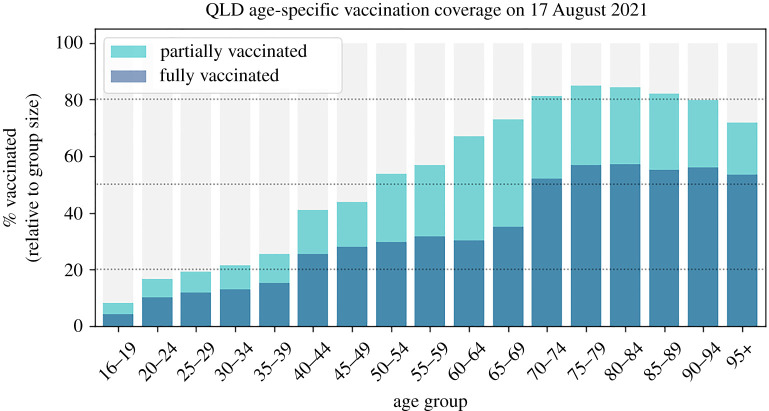
Age-specific state of vaccination in Queensland as of 17 August 2021. The height of each bar represents the percentage of people that have been either partially or fully vaccinated in that age group. Dotted lines indicate 20%, 50% and 80%. (Online version in colour.)

The key empirical inputs to the model used in this work include: (1) the composition of age and sex of the Queensland population; (2) testing rates, tracing delays, and isolation and quarantine compliance representative of the period from February to October 2021; and (3) the status of the vaccination roll-out as of the middle of August 2021. The demographics of Queensland were taken from the 2016 census by the Australian Bureau of Statistics (reference period September 2020). We do not include additional public health interventions (beyond TTIQ) such as short lockdowns or layer-specific reduction in transmissibility due to mask wearing, so as to understand what would be the epidemic trajectories that could eventuate if any one variant of the virus is allowed to spread. We note that testing and contact-tracing interventions used here (reflecting 2021 conditions) have changed with respect to the 2020 period for which the Queensland model was originally calibrated [33]. The average number of daily tests increased from around 6200 to approximately 7500. This new baseline was estimated by calculating the mean number of tests between 15 February and 11 May 2021 using data from https://covidlive.com.au/report/daily-tests/qld (last accessed 17 February 2022). The vaccination data used to determine the ‘incomplete’ status of the vaccine roll-out (figure 2) were obtained from the daily reports from Australian Department of Health data. The vaccination roll-out status on 17 August 2021 was the following: approximately 43% partially vaccinated people (having received at least one dose) and 25% fully vaccinated people (having received two doses) older than 16 years, with lower rates for the young for whom access to vaccines began later.

### (c) General design of outbreak scenarios

To determine whether herd immunity can be reached, we need to simulate outbreak scenarios. By using the calibrated Queensland model [33], we explored the impact of ‘introduction scenarios’ in which a fixed number of infections were released into a population of 200 000 agents, a fraction of which had already been vaccinated. In all the scenarios presented here, we infect 20 agents; this number of imports is all but guaranteed to generate sustained transmission without extinguishing by chance in the absence of further controls [33]. The agents making up the group of imported cases are drawn randomly and independently (i.e. they do not necessarily belong to the same household or workplace, though they could be related). This approach enables us to model scenarios such as an undetected leak from hotel quarantine (a common border control in Australia for much of the pandemic) or interstate travel, non-compliance with self-isolation, or separate undetected clusters in the community. A group of imported cases has either the Alpha variant or the Delta variant, but not a mix of both.

All simulations included contact tracing (assuming no limit on the number of contacts traceable per day), testing, and an assumed near-perfect effectiveness of both domestic self-isolation and quarantine on every network except the home network (see electronic supplementary material, table S5). As in previous work, we define quarantine as segregation of potentially infected agents (not to be confused with hotel quarantine) and isolation as segregation of confirmed (via testing) infected agents. When infected agents are detected via testing, they are put in isolation. As a secondary effect the contacts of confirmed cases are traced and tested (with higher priority than the general population). The exposed contacts are then quarantined while they wait for their test result.

Covasim’s default assumption is that the transmissibility of asymptomatic and symptomatic cases is the same. This assumption is based on the results presented by He *et al.* [46], where they did not find a statistically significant difference between transmissibility in symptomatic and asymptomatic cases. Further, while people with symptomatic disease may have higher viral loads and thus be more infectious, people who are actively experiencing symptoms are also less likely to be going about their normal daily activities (even if they have not been tested). We do not know the magnitude of either of these opposing effects, so by default we leave the relative transmissibility of asymptomatic and symptomatic cases at 1. We used this same assumption for our simulations with the Alpha, Delta and Omicron variants.

### (d) Modelling vaccination

We modelled a range of levels of vaccine coverage, that is, the *vaccinated proportion*, representing different stages (or stalling points) of the vaccination roll-out to the eligible population. Unless specified otherwise, the eligible population consists of people aged 16 and older to represent Queensland’s eligible population in August 2021. In Australia, the Pfizer–BioNTech vaccine was approved for children aged 12–15 years on 13 September 2021 [47] and for children aged 5–11 years on 10 January 2022 [48]. We modelled vaccination as affecting individual immunity in two distinct ways, each approach highlighting the effects of vaccination as relevant to the question at hand.

#### (i) Simple vaccination model

The first approach to vaccination [45] is a simplified implementation that directly modulates the likelihood of becoming infected on contact with an infected individual, and blocks progression to symptomatic disease for people who are vaccinated and become infected. This approach has one vaccine parameter: vaccine effectiveness against infection, representing a reduction in the probability of acquiring an infection when a contact occurs with an infectious case. This vaccination model reflects the number of completed (two-dose) vaccine treatments assigned to randomly selected members of the eligible population. We note that the vaccine’s protection against infection can be thought of as leaky [49], not sterilizing (i.e. it is not the case that 80% effectiveness means that 80% of vaccinated people have perfect protection and 20% have no protection). Using this vaccine model, each person vaccinated has a reduced but non-zero risk of becoming infected based on the vaccine effectiveness, with the only exception to this rule being a vaccine effectiveness of 100%, which reduces the probability of acquiring an infection to 0. This vaccine model makes the additional assumption that people who are vaccinated and become infected will be 100% protected from symptomatic disease, meaning that they will no longer develop symptoms (both mild and severe) or progress into critical states and death (see electronic supplementary material, table S7). Another simplifying assumption of this model is that it does not explicitly model the reduction of onward transmission [50–52]. We used the simple vaccine model in the simulated scenarios presented in Case 1a in the next section, where we systematically explore the levels of protection against outbreak afforded by combinations of two parameters: vaccine effectiveness and vaccine coverage (reported as a fraction of the eligible population vaccinated).

#### (ii) Realistic vaccination model

The second approach to vaccination [31] moderates each agent’s relative susceptibility to infection and to developing symptoms (i.e. asymptomatic versus symptomatic) and their severity of symptoms (pre-symptomatic, mild, severe needing hospitalization, and critical needing intensive care). This approach also accounts for protection against infection, protection against onward transmission (approx. 40% [50,51]), protection against symptomatic disease and protection against severe disease [31] (see electronic supplementary material, table S7). Further, with this vaccination approach, it is possible to have agents that have received only one dose (i.e. a partial vaccine treatment) and agents that have been vaccinated with two doses (i.e. a full vaccine treatment). The ideal interval between receiving the first and second doses is determined by the vaccine brand. The most important feature of this second approach is that it uses a detailed mechanistic model of immunity [31]; therefore, it can also account for waning vaccine effectiveness over time, i.e. the decay in relative susceptibility to infection due to a decay in neutralizing antibody concentration following vaccination. We employed this vaccination model for the scenarios presented in Case 1b in the next section, where we systematically assess whether herd immunity is achieved for combinations of two parameters—partial versus full vaccination coverage—and in Case 2, where we assess the effect of age-specific hesitancy in health outcomes. In Cases 1b and 2, all vaccinated agents received the Pfizer–BioNTech vaccine, and waning immunity was accounted for.

In this realistic vaccine model [31], protective efficacy is modelled as a function of neutralizing anitbodies (NAbs) [53]. Naive individuals are assumed to have no protective NAbs against SARS-CoV-2. Upon vaccination or infection, individuals draw an initial NAb level from a lognormal distribution, following the distribution used in the study by Khoury *et al.* [53]. For vaccine-induced immunity, the specific parameters of this distribution depend on the vaccine administered and have been reported in the study by Cohen *et al.* [31]. The key assumptions underlying NAb kinetics are the following: NAbs grow linearly until they reach their peak after three weeks and then follow a two-part exponential decay, with a 100-day half-life in the first 250 days and an exponentially decaying decay rate until a 10-year half-life is achieved [31,53]. The implementation in Covasim allows for customization of the functional form of the immunity kinetics, but because this is not the main focus of the present work, we assume that all individuals have the same kinetics for naturally acquired and vaccine-induced immunity.

### (e) Modelling hesitancy

**Figure 3.**
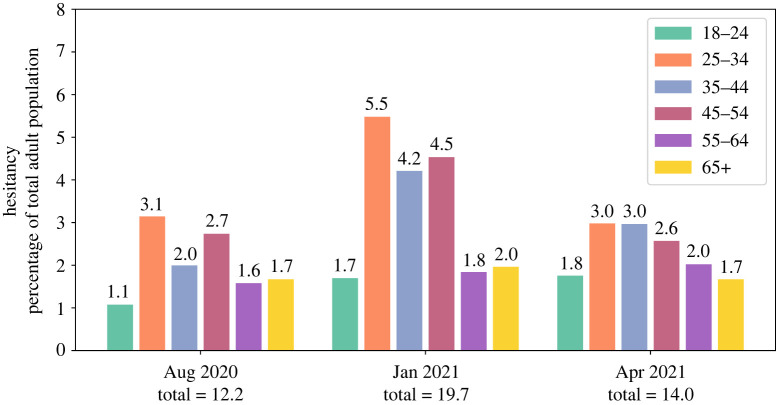
Empirical Australian hesitancy rates in the adult population [26] in August 2020, January 2021 and April 2021, for six representative age groups. In this plot, the height of each bar is the hesitancy rate of a specific age group, and its value is expressed as a percentage relative to the size of the total adult population (i.e. aged 18 years and over). (Online version in colour.)

Hesitancy is not uniform across age groups, with increased hesitancy in the young, as verified from survey data collected from Australian participants in August 2020, January 2021 and April 2021 [26] (figure 3). Indeed, between August 2020 and January 2021, hesitancy increased for all age groups but with a much larger increase for young adults than for the elderly. Thus, we modelled the effects of age-specific hesitancy by first selecting the proportion of agents who are hesitant, and leaving them unvaccinated for the course of the simulation. We used empirical data on hesitancy stratified by age [26] and generated high, low and optimistic hesitancy levels derived from observed variability between August 2020 and January 2021. In addition, we investigated whether the age structure of hesitancy matters beyond the overall level of hesitancy, by setting the level of hesitancy to be homogeneous across all agegroups.

### (f) Assessing herd immunity and health outcomes

To assess whether the population has achieved herd immunity, we used the effective reproductive number ($r_{\mathrm{eff}}$). This number encompasses the (average) number of secondary cases per infectious case in a population with multiple levels of susceptibility and/or immunity (derived naturally following infection or from vaccination). In Covasim, the default method for computing $r_{\mathrm{eff}}$ is as follows:

2.1 $$r_{\mathrm{eff}}(t)=\frac{I_{n}(t)}{I_{a}(t)}\;w(t),$$

where $I_{n}(t)$ is the number of new infections on day t, which is divided by $I_{a}(t)$, the number of actively infectious people on day t, and multiplied by the mean duration of infectiousness w(t). These instantaneous $r_{\mathrm{eff}}$ estimates are smoothed using a seven-day window.

The control threshold of $r_{\mathrm{eff}}=1$ is used to assess whether herd immunity has been achieved. If $r_{\mathrm{eff}}>1$, the number of new infections is increasing, and if $r_{\mathrm{eff}}<1$, there will be a decline in the number of new infections and the spread will eventually stop. Because we want to capture the undiluted effectiveness of vaccination interventions, $r_{\mathrm{eff}}$ needs to be measured within a period where herd immunity has not been reached through widespread naturally acquired immunity. Further, we also take into account other practical considerations to narrow down the date on which we would assess herd immunity. For instance, within the first week of a simulation, $r_{\mathrm{eff}}$ estimates may have fluctuations [54] because of the underlying transient dynamics of the model and the padding used for smoothing $r_{\mathrm{eff}}$ [55]. Thus, we did not consider the first week as a suitable assessment period. We also let the epidemic trajectory evolve a further two weeks to account for the delays in becoming infectious, onset of symptoms and reporting. After all, $r_{\mathrm{eff}}$ at time t still reflects transmission events from some time in the past. On the basis of these considerations and preliminary results, we picked an observation window between days 21 and 35, where $r_{\mathrm{eff}}(t)$ was relatively constant or its derivative close to 0 (see electronic supplementary material, figure S1). Thus, for each simulation, we extracted the value of $r_{\mathrm{eff}}$ 30 days after introducing 20 infected agents carrying a particular variant, either Alpha, Delta or Omicron, but not a mix of them. In the figures in §3, we present the mean of this value, averaged across 1000 simulations and simply denoted by $r_{\mathrm{eff}}^{30}$.

To assess herd immunity for Case 1a and Case 1b in §3*,* we define Queensland’s critical vaccination threshold to be the fraction of the eligible population that needs to be fully vaccinated so that $r_{\mathrm{eff}}^{30}=1$. However, to account for the uncertainty across the finite samples of 1000 simulations each, we determine a range of thresholds by defining bounds using the standard error in $r_{\mathrm{eff}}^{30}$ (${\text{SE}}_{r_{\mathrm{eff}}^{30}}$ shown for every scenario of Case 1a in the electronic supplementary material, fig. S2). Therefore, the range of critical vaccination thresholds encompasses vaccine coverage values between (1) the highest vaccine coverage level for which $r_{\mathrm{eff}}^{30}-{\text{SE}}_{r_{\mathrm{eff}}^{30}}>1$ (upper coverage bound) and (2) the lowest coverage level for which $r_{\mathrm{eff}}^{30}+{\text{SE}}_{r_{\mathrm{eff}}^{30}}<1$ (lower coverage bound).

To assess the effect of hesitancy in Case 1a in §3, we consider the cumulative number of deaths, the cumulative number of infections and critical cases (used as a proxy for ICU occupancy) as representative health outcomes to be minimized. We report these quantities in the form of boxplots to give an overview of typical values and the uncertainty in each scenario.

## 3. Results

### (a) Case 1: What are the requirements for Queensland to reach herd immunity?

#### (i) Case 1a: impact of protection against infection, and blocking symptomatic COVID-19 using a simple vaccination model

**Figure 4.**
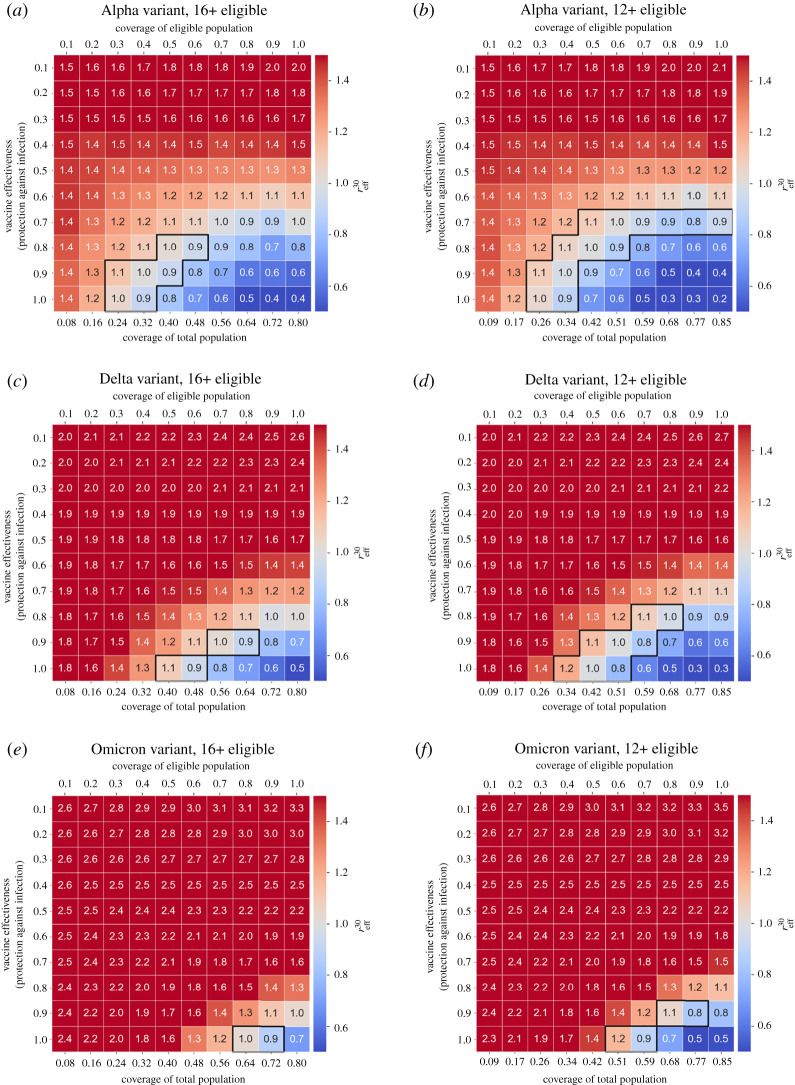
Effective reproductive number 30 days after seeding an outbreak ($r_{\mathrm{eff}}^{30}$), as a function of vaccine effectiveness (infection blocking) and vaccine coverage (fraction of the eligible population vaccinated, shown along the top axes). The fraction of the total population vaccinated is shown along the bottom axes. Outlined areas indicate values of vaccine coverage over which herd immunity is reached as per the definition given in §2f for those vaccine effectiveness values at which herd immunity is achieved at all. We have clipped the colour scale such that $r_{\mathrm{eff}}^{30}\in$ [0.5, 1.5] to better see the zone near $r_{\mathrm{eff}}^{30}=1$. (*a*) Alpha variant, ages 16+ eligible; (*b*) Alpha variant, ages 12+ eligible; (*c*) Delta variant, ages 16+ eligible; (*d*) Delta variant, ages 12+ eligible; (*e*) Omicron variant, ages 16+ eligible; (*f*) Omicron variant, ages 12+ eligible. Initial number of imported infections is 20, and other simulation parameters are summarized in the electronic supplementary material, table S6. (Online version in colour.)

We examined the combined effect of vaccine coverage and vaccine effectiveness (protection against infections) on outbreaks where only one of the main variants (Alpha, Delta or Omicron) was dominant. With the eligible population restricted to ages 16 and above, we found that reliably controlling outbreaks is substantially more difficult for Delta than for Alpha (figure 4*a*,*c*) and unrealistic for Omicron (figure 4*e*). Reliably achieving herd immunity (with TTIQ in place) against the Alpha variant requires vaccine effectiveness of at least 80% with a minimum of 60% coverage of the eligible population (48% of the total population), and at least 50% coverage (40% of total population) for 90% effectiveness. Against the Delta variant, herd immunity is only reliably achieved for a vaccine effectiveness of 90% or above and requires at least 90% vaccination coverage of the eligible population (i.e. approx. 72% of the total population). Herd immunity can only be achieved against the Omicron variant if the vaccine provides 100% protection against infection and at least 90% of the eligible population is vaccinated (at least 72% of the total population). The values we just mentioned are the upper bounds of requisite vaccination coverage, which show the lowest level of uncertainty (i.e. more than 50% of the simulations have $r_{\mathrm{eff}}^{30}<1$ as shown in the electronic supplementary material, fig. S3).

We repeated these scenarios for an expanded eligible population including individuals aged 12 years and older (this expansion of eligibility occurred in Queensland in September 2021) (figure 4*b*,*d*,*f*). These results show that herd immunity may be achieved against Alpha for a vaccine coverage as low as around 40% of the eligible population (approx. 34% of the total population), assuming a vaccine effectiveness of at least 90%. Against Delta, herd immunity may be achieved with a vaccine effectiveness of at least 80% and vaccine coverage of 80% of the eligible population (at least 68% of the total population). Herd immunity is not attainable against Omicron unless the vaccine effectiveness is at least 90% and 90% of the population aged 12 and older is vaccinated (approx. 77% of the total population).

We also repeated these scenarios for when the entire population is eligible for vaccination (electronic supplementary material, fig. S8 for mean $r_{\mathrm{eff}}^{30}$; figs. S9 and S10 for uncertainty measures of $r_{\mathrm{eff}}^{30}$). These results are consistent with those for an eligible population aged 12 and older: for a vaccine with 90% effectiveness against the Alpha variant, approximately 35% of the total population would need to be vaccinated. For a vaccine with 90% effectiveness against Delta, at least 50% of the population would need to be vaccinated. Herd immunity can also be achieved against the Omicron variant with a 90%-effective vaccine if at least 70% of the population is vaccinated, slightly lower than both the 90% eligible and 77% total population required if only those aged 12 and older are vaccinated. These results support the idea that the expansion of the vaccination programme can have a marked qualitative impact on the dynamics of the spread against Omicron.

As a secondary effect, we note that for low vaccine effectiveness, the $r_{\mathrm{eff}}^{30}$ values increase for increasing coverage (top right corner of figure 4*a*–*d*). This is because this simplified vaccine model assumes perfect protection against symptomatic disease, which interacts with differential testing rates for asymptomatic and symptomatic individuals. In essence, this models the effect of ‘silent spreaders’, overconfident that they are protected and less likely to test.

Table 1 summarizes the critical levels of vaccination needed in Queensland for practical herd immunity against the Alpha or Delta variants (maintaining testing, testing and isolation measures), for values of vaccine effectiveness against infection of 80% or higher and all three eligible populations shown in figure 4 and the electronic supplementary material, fig. S8.

**Table 1.** Critical vaccination threshold ranges necessary to reach herd immunity in Queensland against the Alpha, Delta and Omicron variants, given a vaccine effectiveness of 80%, 90% or 100%, respectively; n.a. means herd immunity is not achieved.

vaccine effectiveness	eligible population		
0.8 (80%)	16+	12+	0+
Alpha	50–60%	40–60%	35–45%
Delta	n.a.	70–80%	60–65%
Omicron	n.a.	n.a.	85–90%

0.9 (90%)	16+	12+	0+
Alpha	30–50%	30–40%	30–35%
Delta	70–80%	50–70%	50–55%
Omicron	n.a.	80–90%	65–70%

1.0 (100%)	16+	12+	0+
Alpha	30–50%	30–50%	25–30%
Delta	50–60%	40–60%	40–45%
Omicron	80–90%	60–70%	55–60%

#### (ii) Case 1b: impact of different coverage rates of single versus double doses using a realistic vaccination model

**Figure 5.**
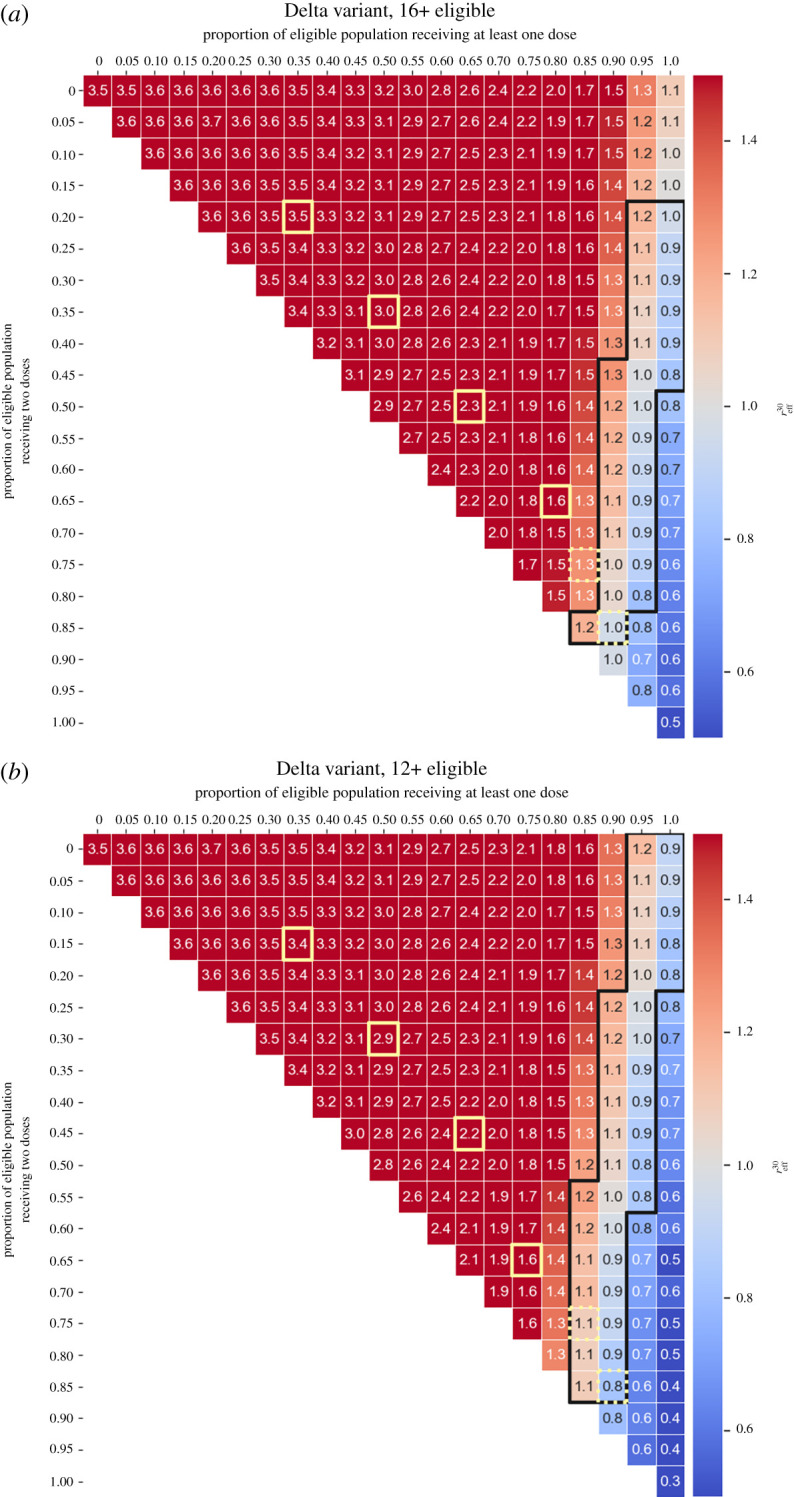
Partial versus full vaccination coverage. Effective reproductive number on day 30 after initial infections are imported ($r_{\mathrm{eff}}^{30}$) as a function of the proportion of people who received at least one dose and the proportion of people who received two doses (everyone received the Pfizer–BioNTech vaccine and the proportions along the top axis are with respect to the eligible population). In all scenarios 20 agents infected with the Delta variant are inserted into the community. We have clipped the colour scale such that $r_{\mathrm{eff}}^{30}\in$ [0.5, 1.5] to better see the zone near $r_{\mathrm{eff}}^{30}=1$. (*a*) Eligible population aged 16+; (*b*) eligible population aged 12+. From top left to bottom right, the yellow squares illustrate the progression of the vaccine roll-out in Queensland, starting from 1 August 2021 and proceeding along the first of each month through to January 2022 [56]. (Online version in colour.)

We next asked whether vaccine roll-outs with supply constraints should target the completion of second doses or aim for maximal first-dose coverage first. We simulated scenarios where we vaccinated a proportion of people with only one dose (partial vaccination) and a proportion of people with two doses (full vaccination). In these scenarios, everyone received Pfizer–BioNTech doses, and coverage values are with respect to the eligible population. Vaccine efficacy values used in these scenarios are presented in the electronic supplementary material, table S7. We found that very high ‘at least one dose coverage’ (>95%) confers substantial protection against infection, with $r_{\mathrm{eff}}^{30}\approx 1$ even for low rates of ‘two doses’ coverage (<30%), for eligible populations who are 16+ (figure 5*a*) and 12+ (figure 5*b*).

Using the definition of herd immunity given in §2f, we find that for an eligible population aged 16+, if 100% received at least one dose, herd immunity could be achieved if 25–45% of the eligible population receive two doses. On the other hand, if 100% of an eligible population aged 12+ received at least one dose, herd immunity would be achieved with a lower and narrower two-dose coverage of 0–20%.

**Figure 6.**
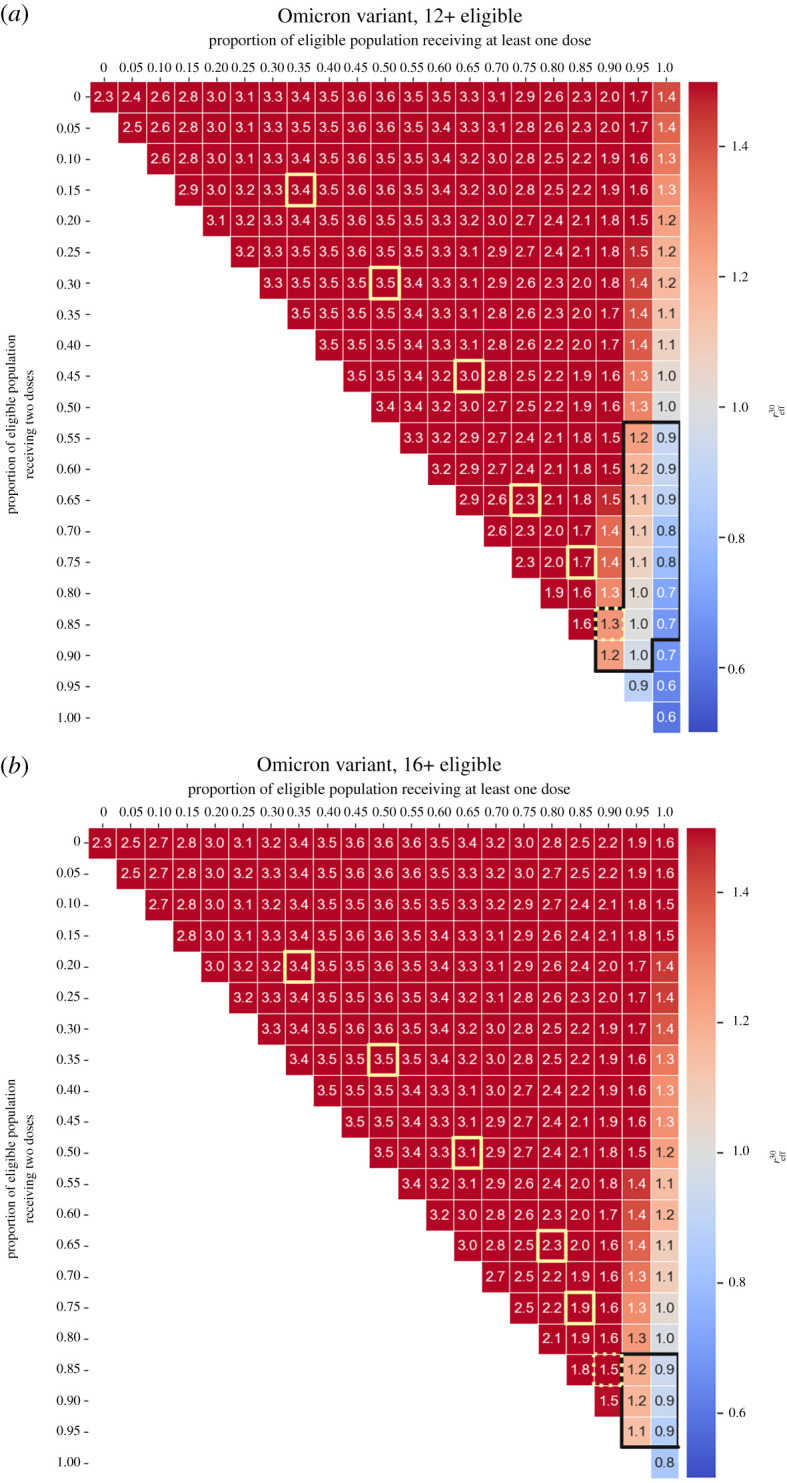
Partial versus full vaccination coverage. Effective reproductive number on day 30 after initial infections are imported ($r_{\mathrm{eff}}^{30}$) as a function of the proportion of people who received at least one dose and the proportion of people who received two doses (everyone received the Pfizer–BioNTech vaccine and the proportions along the top axis are with respect to the eligible population). In all scenarios 20 agents infected with the Omicron variant are inserted into the community. We have clipped the colour scale such that $r_{\mathrm{eff}}^{30}\in$ [0.5, 1.5] to better see the zone near $r_{\mathrm{eff}}^{30}=1$. (*a*) Eligible population aged 16+; (*b*) eligible population aged 12+. From top left to bottom right, the yellow squares illustrate the progression of the vaccine roll-out in Queensland, starting from 1 August 2021 and proceeding along the first of each month through to January 2022 [56]. (Online version in colour.)

We also repeated these scenarios for the Omicron variant, for eligible populations aged 16+ (figure 6*a*) and 12+ (figure 6*b*). These results show that even with a high proportion (>95%) of the eligible population receiving at least one dose, a high proportion of the eligible population should receive two doses to achieve herd immunity: 85–95% (eligible population 16+) and 55–85% (eligible population 12+). These results are consistent with what occurred in Queensland between December 2021 and January 2022. The proportion of the eligible population aged 16+ who had received two doses was less than 80% and put the population in a situation where an outbreak was inevitable. Uncertainty measures of $r_{\mathrm{eff}}^{30}$ for the scenarios presented in figures 5 and 6 are provided in the electronic supplementary material, in figures S4 and S5 and in figures S6 and S7, respectively.

Our results in figures 5 and 6 show that prioritization of two-dose vaccination (i.e. moving down the diagonal instead of moving to the right) would result in minimal reductions in $r_{\mathrm{eff}}^{30}$. These results indicate that administering first doses should be prioritized regardless of the minimal age included in the vaccination programme. However, to control outbreaks by bringing $r_{\mathrm{eff}}^{30}$ down below 1 with TTIQ alone requires administration of second doses—particularly since the one-dose coverage of at least 95% has proven to be difficult to attain in Queensland.

### (b) Case 2: How does vaccine hesitancy affect health outcomes in Queensland?

We next examined scenarios with realistic age-dependent hesitancy rates and the realistic vaccine model (see electronic supplementary material, table S9, for a breakdown of these values by age group). We simulated outbreaks of the Delta variant with TTIQ as the only intervention and observed the resulting epidemic trajectories. We first note that herd immunity was not reached in most of these simulations (i.e. $r_{\mathrm{eff}}^{30}>1$ and large outbreaks occur, albeit occasionally delayed by initial slow growth in a highly vaccinated population). To assess disease severity and burden, in each scenario we quantified infections, critical cases and deaths. We also chose to report cumulative results at 90 days as our cut-off, as these key indicators take time to accumulate.

**Figure 7.**
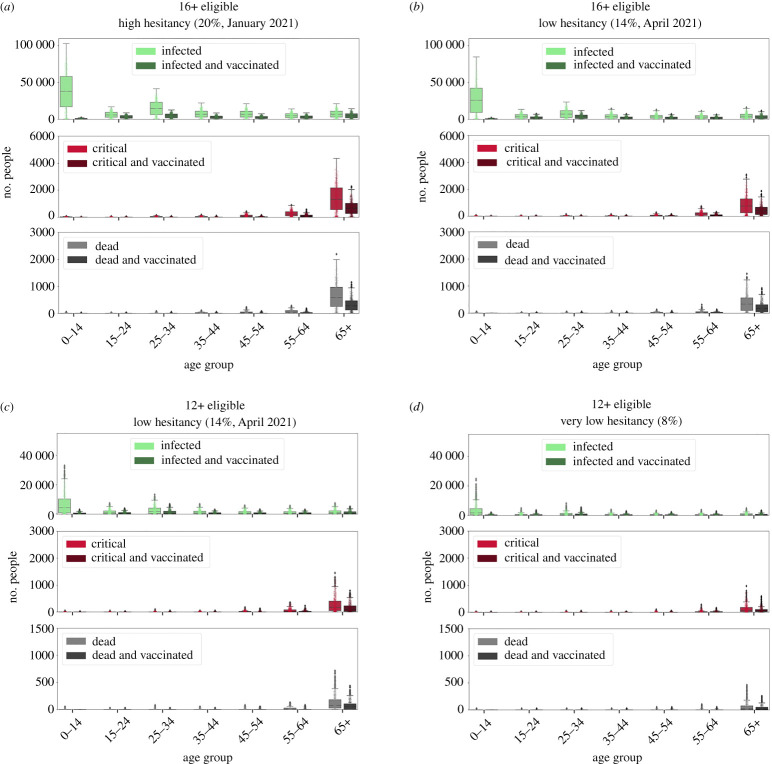
A comparison of age-specific health outcomes: infections (green), critical cases (red) and deaths (grey) 90 days after the insertion of 20 agents infected with the Delta variant. (*a*) Eligible population aged 16+, with age-specific hesitancy as in January 2021. (*b*) Eligible population aged 16+, with hesitancy as in April 2021. (*c*) Eligible population aged 12+, with hesitancy levels as in April 2021 and with individuals aged 12–17 assigned their parents’ hesitancy (i.e. in the 35–44 bracket). (*d*) Eligible population aged 12+ and with all agents having 8% hesitancy, which is the lowest observed in empirical data across all age groups. The values correspond to the numbers of individuals in each state and age bin (boxplot of 1000 simulations, with individual results overlaid), for all individuals (light-coloured bars) and the vaccinated subset (dark-coloured bars). The hesitancy and vaccination coverage are summarized in the electronic supplementary material, table S9. (Online version in colour.)

We asked whether the observed fluctuations in age-specific hesitancy levels in Australia (figure 3) would have a significant effect on health outcomes, assuming that these hesitant proportions represent the final unvaccinated populations. We used values from January 2021 (19.7%) and April 2021 (14.0%), which were representative high and low points during the first half of 2021. We henceforth refer to these levels of hesitancy as ‘high’ and ‘low’. With the delta variant, the change from ‘high’ hesitancy to ‘low’ hesitancy manifests in all age categories as lower numbers of infections, critical cases and deaths (figure 7*a*,*b*). While the $r_{\mathrm{eff}}^{30}$ mean values and standard deviation were similar for the ‘high’ and ‘low’ hesitancy scenarios (e.g. 1.5±0.7 and 1.4±0.8, respectively), small differences in the exponential growth rate can manifest as large differences in health outcomes after 90 days. The largest differences in health outcomes were a 52% decrease in the number of infected people aged 25–34 (attributable to the particularly large difference in hesitancy among the young) and a 43% decrease in the number of deaths for the vulnerable 65+ age group. This demonstrates that a drop from around 20% to 14% in overall hesitancy—with larger drops in the hesitant young—can have a relatively large positive effect on lives saved and burden of disease.

To confirm that the age structure makes a difference beyond simply the overall rates of hesitancy, we compared health outcomes between the empirical age distributions and surrogate distributions with constant non-specific hesitancy (electronic supplementary material, fig. S11). This comparison shows that the total number of infections remain similar (with decreases in the youthful population and increases in the older population), but the age structure contributes to substantial differences in deaths and critical cases. Non-specific hesitancy yields up to 43% more deaths than age-specific hesitancy, and hence the age structure influences outcomes beyond simply the total vaccination coverage.

We next expanded the eligible population to include individuals aged 12 and over and simulated two additional scenarios with plausible hesitancy levels. The first scenario assumed the ‘low’ hesitancy level (14.0%). Agents aged 12–16 were assigned the same hesitancy as the 35–44 age group, which we assumed to be their parents (figure 7*c*). The second scenario set hesitancy homogeneously across all age groups to 8%, which was the lowest observed hesitancy of any age group in April 2021 (figure 7*d*), henceforth referred to as ‘very low’. We note that these scenarios fell within the region where herd immunity could be attained for the Delta variant. Their Delta $r_{\mathrm{eff}}^{30}$ mean values and standard deviations were 1.0±0.8 and 0.9±0.7 for the ‘low’ and ‘very low’ hesitancy scenarios, respectively.

A qualitative comparison between the health outcomes of the scenario with 16+ eligibility and ‘low’ hesitancy (figure 7*c*) and the two 12+ eligibility scenarios with ‘low’ and ‘very low’ hesitancy (figure 7*b*–*d*) reveals that in the latter two, there is a reduction by more than half in the cumulative number of infections, critical cases and deaths in all age categories. Expanding the eligible population to include the 12–15 age group reduced the median number of infections in the 0–14 and 15–24 age groups by over 75%, and by a further 60% if all age groups can reach the vaccination rates of the oldest group (‘very low’ hesitancy). Large improvements were also seen outside of this age group, with vaccination of 12- to 15-year-olds reducing the median number of deaths in the 65+ age group by 77% and by a further 66% for ‘very low’ hesitancy case.

## 4. Discussion

In this work, we aimed to (i) determine whether plausible combinations of vaccine effectiveness and vaccine coverage in Queensland allow the possibility of reaching herd immunity against the Alpha, Delta and Omicron variants, without widespread natural immunity; and (ii) determine whether age-dependent variability in hesitancy, coupled to the population age structure, poses additional challenges for control of COVID-19 outbreaks. The main rationale was to understand whether vaccination and mild non-pharmaceutical interventions such as TTIQ would be sufficient to prevent outbreaks given the rise of new variants. We further expected that identifying age brackets that have the largest impact on health outcomes may help to guide strategies for boosting vaccine willingness in key demographics.

Our modelling results highlight how important it is that Queensland, and other settings with similar demographics and low natural immunity, vaccinate its population with high coverage. We identified practical critical vaccination thresholds for protection against the Alpha and Delta variants: for Alpha, only 48% of the overall population is required to be vaccinated to drastically curb the number of deaths and infections, this lying at the upper edge of the range of critical thresholds; with the Delta variant, 48% coverage lies at the lower edge of critical vaccination thresholds, and herd immunity can be reliably achieved only if 72% of the total population is vaccinated. These Delta targets were only reached around the end of 2021, by which time the Omicron variant had already arrived. But in any case once we factor in waning of immunity—equivalent to weakening of vaccine effectiveness in our simulations—herd immunity cannot be reached. Hence, Queensland is unlikely to be sufficiently protected against new incursions without additional health measures beyond TTIQ. This situation can only be worsened by waning compliance with control measures in the population as the pandemic drags on.

In terms of health outcomes, results comparing different levels of hesitancy demonstrate that a small drop in global hesitancy (from around 20% to 14%), within the ranges that have been recorded in longitudinal surveys, can greatly reduce infections and deaths. Moreover, we showed that more extensive vaccination of the young can protect the elderly. Reducing hesitancy of the population aged 12–34 years can greatly reduce deaths in older age brackets. Our results also support targeting high vaccine uptake among the group aged 12–15; school-based programmes may be beneficial for achieving this. Further cross-interactions between age-specific hesitancy and different population demographics should also be investigated (e.g. cross-interaction between age-specific hesitancy and use of social media, or age-specific hesitancy and geographic location). It also remains to be seen how adult hesitancy will affect consent for their children to receive vaccines.

One limitation of this study is that we have not sought to model the roll-out trajectory itself, instead opting for the simpler case of examining the situation once the roll-out stalls upon vaccinating all of the (currently projected) willing members of the population. We did, however, examine differences in $r_{\mathrm{eff}}$ between different rates of single versus double doses (which can be important [4,41,57]). We also simplified the mix of vaccines, modelling scenarios that assumed an overall level of vaccine effectiveness averaged across vaccine brands, as well as optimistic scenarios where all agents were vaccinated with Pfizer–BioNTech, whose effectiveness may be overestimated with respect to Omicron. In the Pfizer–BioNTech-only simulations, the waning effectiveness for all agents started at the same date—creating a relatively homogeneous level of protection in different age groups. The specific vaccine brands and their waning effectiveness in each age group is an area for future work. Our results spanning a range of effectiveness values (figure 4) speak to possible waning scenarios: waning corresponds to reduced effectiveness and hence to drifting upwards in the plotted grid. Roll-out of vaccine booster doses can be considered as undoing the waning, corresponding to rapidly moving downwards in the plotted grids. Likewise, the potential effects of new variants (e.g. Omicron and beyond) could be mapped out systematically by exploring different rates of vaccine escape and intrinsic transmissibility.

Another simplification in our scenarios was the assumption that only one variant was circulating at any time, rather than a mix of multiple variants. At the time this work was carried out, our assumption was supported by earlier modelling studies which had shown that in settings like Queensland with near-zero prior naturally acquired immunity and no countermeasures in place, a more transmissible variant (like Omicron) rapidly outcompetes a less transmissible variant (like Delta) [30,31,58]. This assumption was confirmed by the Omicron wave experienced in Queensland in January 2022. When Queensland reopened its interstate borders in the middle of December 2021, Delta was the prevalent variant in Australia, but by the end of December 2021 it was estimated that Omicron (BA.1) accounted for approximately 84% of infections [59]. Indeed BA.1 has since been usurped by BA.2 (as of May 2022), and there are other even more transmissible Omicron subtypes in circulation globally [60].

Other limitations that would likely affect the transmission dynamics include our assumption that TTIQ effectiveness does not reduce over time, which would reflect ‘fatigue’ with restrictions in the community as well as the rolling back of such interventions (and indeed as of May 2022 contact tracing has largely ended in Queensland and close contacts no longer need to isolate in most cases). We also do not model seasonal or day-of-the-week effects on contact networks. While we have incorporated a moderate degree of realism in our contact networks, there remains considerable uncertainty in their parametrizations, and particular high-risk settings (aged care, hospitals and their workforces) are not explicitly modelled.

Beyond the short term, demographic changes could affect the course of the pandemic: changes in age distribution and household size and composition have been shown to be significantly associated with different rates of COVID-19 incidence and mortality [61,62]. Given that Australia’s large ‘baby boomer’ generation is approaching the ages at which risk of severe outcomes increases steeply, and coupled with waning immunity, concerted effort may be required to maintain immunity levels to prevent deterioration of health outcomes. Moreover, births supply a steady stream of new unvaccinated individuals faster than the death rate. While Australia has a generally high vaccination rate for children, and high coverage overall against COVID-19, only about 40% of Queensland children aged 5–11 have received their first dose as of May 2022. Sustained low coverage among children could thus erode total population vaccination coverage, though, in the end, the Omicron wave has conferred substantial immunity to the young.

In this work, we have analysed the level of vaccine coverage and effectiveness to achieve herd immunity in a population with low natural immunity, and while our model has been customized to the population of Queensland and incorporates a high degree of population heterogeneity (e.g. age-specific prognoses and immunity properties [63]), there were many simplifying assumptions made in Case 1a discussed in §3, including the lack of an explicit reduction in onward transmission. However, there is evidence showing that immunization with either the Pfizer–BioNTech [50,51] or the Oxford–AstraZeneca [50] vaccine reduces the chance of onward virus transmission by 40–60%. For that reason, we introduced this effect into our more realistic scenarios—Case 1b and Case 2. We note that despite the absence of onward transmission effectiveness, the results in Case 1a (Delta variant, eligible population aged 12+ and vaccine effectiveness of 90%) yield more optimistic estimates of herd immunity thresholds than those in Case 1b (same variant, same eligible population). This is because in Covasim, a reduction in the probability of developing symptoms results in a reduction in the relative transmissibility of individuals. Because our simple vaccination model provides a perfect protection against symptoms, not only does it counteract the absence of an explicit onward transmission effectiveness but it likely overestimates the overall effect of vaccines on transmission.

The results from our scenarios with Delta and Omicron, an eligible population encompassing the total population and vaccine effectiveness above 80% are consistent with estimates from earlier modelling work [64,65], where herd immunity thresholds sit at 60–70% of the total population, assuming vaccines are delivered at random and (naive) individuals mix at random. However, it has also been shown that prior exposure to infection can lower the herd immunity thresholds to around 40% [65]. These modelling results are supported by retrospective observational studies reporting that previous infection with SARS-CoV-2 provides stronger and longer protection than vaccine-induced protection in naive individuals [66]. For Queensland and similar settings, this indicates that the population may need to adopt frequent administration of boosters compared to other locations such as the UK [64]. However, since the arrival of Omicron in December 2021, Queensland has experienced two waves of infections, with their peaks around the middle of January 2022 and early April 2022, respectively, with more waves expected during the winter season (March–August 2022) [29]. This will confer the population additional protection acquired through infections. Preliminary results from Qatar estimated that the protection gained from Omicron infection against future Omicron (symptomatic) reinfections is approximately 56% [67]. Thus, the effective herd immunity thresholds of Queensland may be lower than the ones obtained in this study.

Perhaps the most critical and outstanding question is: how do we maintain the state of herd immunity in the long run? Factoring in waning of immunity, which is equivalent here to drifting ‘upwards’ in the heatmaps of figure 4, implies that even if herd immunity were attained, it would be a transient state in the absence of boosters.

## 5. Conclusion

In this article, we have assessed the practicality of achieving herd immunity against the Alpha (B.1.1.7), Delta (B.1.617.2) and Omicron (BA.1) variants of SARS-CoV-2 in the state of Queensland, Australia—a region that until the end of 2021 had negligible rates of infection-acquired immunity, thus making our results a rare glimpse into herd immunity attained solely as the result of vaccination-induced immunity, rather than derived from prior infection or a combination of both. Our simulations show that full herd immunity was unlikely to be achieved for the Delta and Omicron variants from currently available vaccines alone. Assuming that vaccination provides, on average, an effectiveness of 90% against infection—which is at the upper end of observed effectiveness [42,68]—extension of the vaccination programme to children seems essential. Restricting eligibility to ages 16+ requires up to 90% coverage, which proved in February 2022 to be near the maximum attainable given hesitancy rates, which should not be neglected. Indeed, we have shown that small changes in vaccine hesitancy, which have occurred in Australia over short periods of time [26], can have a large impact on the health outcomes for the entirepopulation.

## Data Availability

The data used in this work are publicly available from https://github.com/jxeeno/aust-govt-covid19-vaccine-pdf/blob/master/docs/data/air.csv. Code with the Queensland model and data to reproduce the figures can be found at https://github.com/brain-modelling-group/paper-covid-herd-hesitancy. The data are provided in the electronic supplementary material [69].
